# An investigation of hibernating members from the *Culex pipiens* complex (Diptera, Culicidae) in subterranean habitats of central Germany

**DOI:** 10.1038/s41598-020-67422-7

**Published:** 2020-06-24

**Authors:** Dorian D. Dörge, Sarah Cunze, Henrik Schleifenbaum, Stefan Zaenker, Sven Klimpel

**Affiliations:** 1 Institute for Ecology, Evolution and Diversity, Goethe-University, Max-von-Laue-Str. 13, 60439 Frankfurt/Main, Germany; 2 Senckenberg Biodiversity and Climate Research Centre, Senckenberg Gesellschaft für Naturforschung, Senckenberganlage 25, 60325 Frankfurt/Main, Germany; 3 Hesse Federation for Cave and Karst Research, Königswarter Str. 2a, 36039 Fulda, Germany

**Keywords:** Ecology, Biodiversity, Biogeography, Ecological modelling, Population dynamics

## Abstract

The *Culex pipiens* complex encompasses five species and subspecies of the genus *Culex*. Over time, a multitude of morphologically indistinguishable species has been assigned to this complex with several species being classified as important vectors for different diseases. Some species of this complex hibernate in subterranean habitats, and it has been proven that viruses can survive this phase of hibernation. However, studies focusing on the environmental requirements, ecology and spatial and temporal distribution patterns of mosquitos in underground habitats are sparse. Here, we investigate the main environmental factors and dependencies of *Culex*, considering the number of individuals and survival probabilities in underground habitats during the winter months. Methods. Since the State of Hesse, Germany harbors about 3500 to 4000 subterranean shelters ample availability of subterranean habitats there provides a good opportunity to conduct detailed investigations of the *Culex pipiens* complex. In this study, we identified a sample of 727 specimens of overwintering females within the *Culex pipiens* complex from 52 different underground sites collected over a period of 23 years using qPCR. A complete data set of samplings of hibernating mosquitos from 698 subterranean habitats in Central Germany over the same period was available to study the spatial and temporal patterns and the effect of temperature and precipitation conditions on these hibernating populations using a generalized linear model (GLM). Results. Our qPCR-results show, similar to aboveground studies of mosquitos, that *Culex pipiens pipiens* and *Culex torrentium* occur sympatrically. On the other hand, *Culex pipiens molestus* occurred very rarely. The GLM revealed no shifts in species composition over time, but different preferences for subterranean hibernacula, chemical effects on overwintering populations as well as effects of annual and seasonal mean temperature and precipitation during the active phase from March to November. *Cx. p. pipiens* and *Cx. torrentium* are the most common species within Hessian caves and other underground habitats during winter. They co-occur with different frequency without any patterns in species composition. Weather conditions influence the number of overwintering mosquitos during the activity phase. Depending on cave parameters, the number of mosquitos decreases during the winter months.

## Introduction

Culicidae members belonging to the *Culex pipiens* complex are difficult to distinguish morphologically. The most characteristic features include the male genitalia and the larval siphon^1^. According to Vinogradova^1^ the complex encompasses *Culex pipiens pipiens* var. *pipiens*^2^, its biotype *Culex pipiens pipiens* var*. molestus*^3^, *Culex torrentium*^4^, *Culex pipiens quinquefasciatus*^5^, *Culex pipiens pallens*^6^, and *Culex vagans*^7^. The first three are abundant in Germany. While this complex is comprised largely of mosquitos inhabiting urban areas in temperate climates^8^, *Cx. pipiens*, including its subspecies and biotypes*,* may be the most abundant mosquito species worldwide^9^.

Like many other mosquito species, members of the *Culex pipiens* complex transmit different arthropod-borne viruses (arboviruses). Notable is the West Nile Virus (WNV), which has triggered fatal infections and epidemics in Eastern and Central Europe^10–12^ and is also known in Asia, Australia, Africa, the Caribbean and North America^12^. Several studies show that *Culex* species are competent vectors^13–15^. This may also be true for *Cx. torrentium* which is widely distributed in Central Europe^13,14^. Since *Cx. p. pipiens* is ornithophilic^15,16^, it plays a major role in the transmission of WNV within wild bird populations, except in the northern Central and Mid-Atlantic United States, where it shows higher than usual affinity for humans and becomes a bridge vector^17^. Andreadis^17^ attributes this alteration of host preference to potential genetic ancestry with *Cx. p. molestus* and is considered analogous to the assumed hybridization^18^. *Cx. p. molestus* is known to be mammophilic^19^ and found to have a very different behavior compared to *Cx. p. pipiens*. However, *Cx. p. molestus* has shown no difference in feeding behavior compared to *Cx. p. pipiens*^16^ in a survey area in western Portugal. Today, it is commonly accepted that *Cx. p. molestus* is adapted to a subterranean environment^20–23^ and is autogenous (requiring no blood meal prior to its first oviposition, due to a higher nutrient supply during the larval stage), stenogamous (mating in confined spaces) and homodynamous (non-diapausing). On the other hand, *Cx. p. pipiens* is anautogenous (requiring a blood meal prior to its first oviposition), eurygamous (mating in open spaces) and heterodynamous (diapausing)^1,20,21^. The general autogeny and stenogamy of *Cx. p. molestus* and the anautogamy and eurygamy of *Cx. p. pipiens* respectively has been demonstrated in a breeding experiment^23^. *Cx. p. pipiens* is often seen as a subterranean form of *Cx. pipiens*^19,22,24,25^ and could therefore be much more abundant in caves than in contrasting epigean habitats. Hybrids of *Cx. p. pipiens* and *Cx. p. molestus* were proposed to exist and could serve as bridge vectors for arboviruses from birds to humans since they would show a feeding strategy including mammals and birds^26^. Hybrids of *Cx. p. molestus* and *Cx. p. pipiens* may therefore, play a key role in the distribution of certain zoonotic diseases such as WNV.

Depending on the species, mosquitos can survive winter in all three life stages^27^. Either eggs survive the cold season on dry ground, usually in floodplains, and hatch as soon as temperatures rise and a sufficient amount of water is available, or they overwinter as hatched larvae under the ice cover of low waters. Diapausing or hibernating females in underground systems such as caves or mines is the third option.

It is generally assumed that inseminated female *Cx. p. pipiens* hibernate^25,28^ while *Cx. p. molestus* does not need to^29–31^. Depending on the environmental conditions, the lack of diapause of *Cx. p. molestus* may occur either as expressed or as suppressed homodynamy^32^. According to Kjærandsen^33^, *Cx. pipiens* hibernates in caves and cave-like environments, however, the author did not distinguish between *Cx. p. pipiens* and *Cx. p. molestus* in his study. Caves are considered thermally insulated systems^34^, a frequent and established point of view, corroborating Barr^35^ in that the temperature in a cave is constant and close to the average annual temperature of the surrounding region. Caves are divided into three ecological zones: the entrance zone, twilight zone and depth zone^36^. Caves and other subterranean habitats not only have a relatively constant temperature mostly fluctuating in the entrance region, but also have a generally constant humidity gradient. There are several different categories of caves, ranging from caves that have running water to almost completely dry ones^37^. According to Buffington^38^, cave humidity is not a determining factor for choosing a site for diapausing. However, extensively tested reactions of *Cx. fatigans* to different temperatures and humidity levels could prove the avoidance of subterranean habitats with greater than 95% and below 40% relative humidity^39^.

Considering the still unresolved structure of the *Culex pipiens* complex as well as the variability in their biological interactions and lack of knowledge within Germany, this is the first study to include hibernating mosquitos on a larger scale. Hesse is particularly suited to study the population structure and hibernation preferences of *Culex* due to its many subterranean habitats, the wide-ranging distribution of various *Culex* species and the temperate Central European climate in this region of Germany.

We examined the co-occurrence of the three *Culex pipiens* complex species present in Germany and tested whether spatial patterns within the study area occur. Furthermore, we examined if temperature and precipitation conditions in the preceding activity phases influence the number of mosquitos found during winter. We additionally investigated whether the abundance within the subterranean shelters decreases over the winter months and if this temporal pattern is dependent on certain subterranean parameters.

## Material and methods

### Sample material

**Figure 1:**
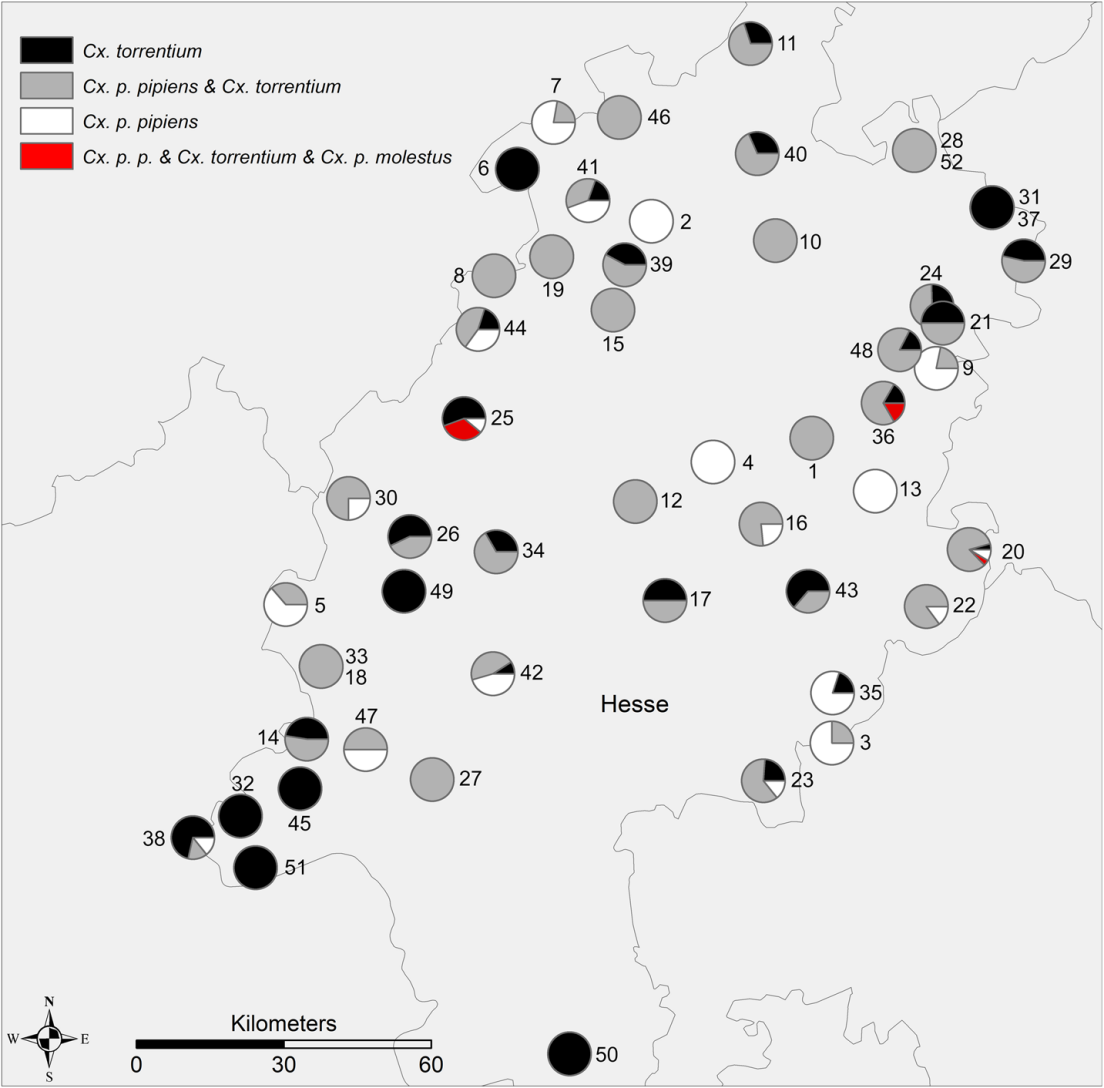
Overview of genetically assessed sample material. Numbers refer to the shelter numbers shown in Table 1. Figure created with ArcGIS Version 10.7^46^.

**Figure 2:**
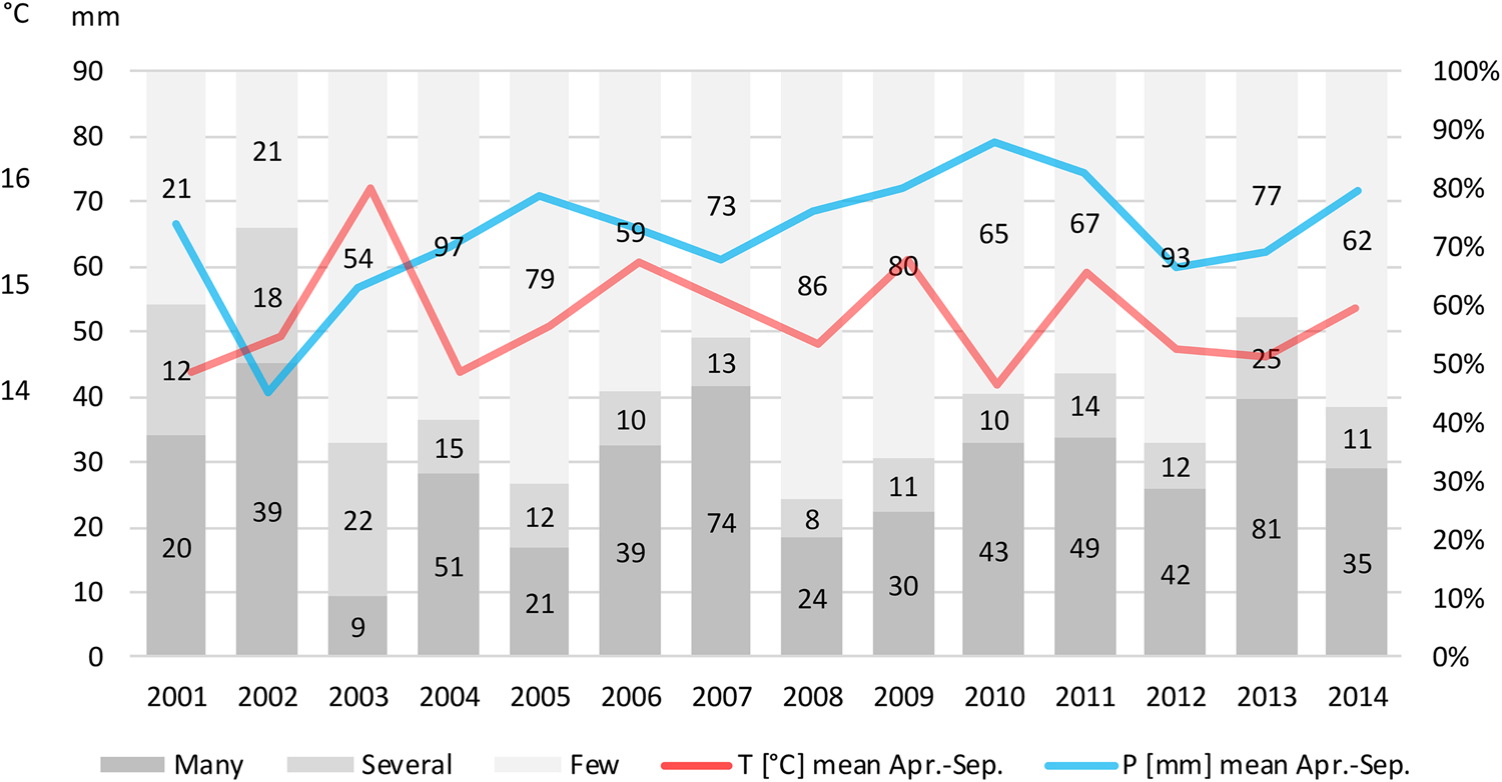
Abundance of hibernating mosquitos in comparison with the climate conditions during the previous activity phase. X-Axis: Year, left Y-Axis: temperature and precipitation, right Y-Axis: composition of categories within the sampling. The numbers in the bar graphs show the absolute frequency of categories in the respective years. Categories: many: > 20 individuals, several: 10 to 20 individuals, few: < 10 individuals found within the subterranean shelter.

A data set consisting of 1827 samples from 698 underground sites served as the basis for our investigations. A total of 8750 mosquitos from the *Culex pipiens* complex were collected from walls and ceilings of subterranean shelters by the Hesse Federation for Cave and Karst Research. Samples were collected during all months throughout the years 1991 to 2014 with a strong focus on winter months in caves, tunnels, cellars and other subterranean shelters. Collection was implemented during the regular inventories of Hessian underground structures. All samples were stored in small, 100% ethanol-filled vials at room temperature until further examination. Specimens collected the same day in the same subterranean shelter were stored together in one vial and labelled as one sample accordingly. We genetically examined a subsample of 727 mosquitos from 52 of the 698 available sites (Table 1) and employed a modified version of the real-time qPCR (Table 2) developed by Rudolf et al.^40^ to gain comparable results. Regarding temporal and spatial patterns of the species’ distribution, the whole dataset of 1827 samplings and species counts from 698 subterranean shelters was used. The spatial distribution patterns of the species compositions are shown in a Gis map (Fig. 1). The temperature and precipitation ratios of the years 2001 to 2014 are shown in comparison with the abundance distributions of the respective years (Fig. 2).

**Table 1:** Spatial pattern analysis (BB = brick-built, BMC = bridge maintenance chamber) of subterranean sites.

Nr	Shelter Type	Humidity	Dec. N	Dec. E	Sampling years
1	Concrete tunnel	Moist	50.7967	9.5485	2007, 2013
2	BB tunnel	Very dry	51.1948	9.0862	2011
3	BMC	Dry	50.2387	9.5996	2011, 2013
4	Rock cellar	Moist	50.7540	9.2631	2008
5	Natural cave	Medium	50.4896	8.0363	2010
6	Mine shaft	Wet	51.2896	8.6955	2006, 2008
7	Touristic mine	Moist	51.3750	8.8005	2004, 2006
8	Rock cellar	Moist	51.0945	8.6287	2004
9	BMC	Moist	50.9221	9.9102	2003, 2006
10	Bunker in quarry	Dry	51.1585	9.4466	2006
11	Bunker complex	Moist	51.5189	9.3776	2006,
12	Rock cellar	Dry	50.6820	9.3776	2009
13	BB cellar	Medium	50.6987	9.7299	2003
14	Mine shaft	Medium	50.2439	8.1010	2008, 2011
15	Rock cellar	Medium	51.0323	8.9745	2001, 2005
16	Rock cellar	Wet	50.6401	9.4005	2007, 2008
17	Rock cellar	Wet	50.5010	9.1237	2008, 2010
18	Mine shaft	MEDIUM	50.1673	9.3542	2005, 2006
19	Rock cellar	Wet	51.1297	8.7965	2001, 2014
20	Rock cellar	MOIST	50.4867	9.8731	2003, 2004, 2005, 2014
21	Mine shaft	Dry	51.0362	9.9002	2002, 2004, 2005, 2006, 2013
22	Rock cellar	Medium	50.5900	9.9984	from 2003 to 2014
23	BB cellar	Dry	50.4286	9.7630	2010
24	Natural cave	Medium	50.1705	9.4033	2001, 2007, 2010, 2011
25	Mine shaft	Medium	50.8330	8.5444	2003, 2009
26	Mine shaft	Medium	50.6164	8.3905	2005
27	Rock cellar	Medium	50.1720	8.4602	2011
28	Mine shaft	Moist	51.2733	9.8713	1994, 2004, 2005, 2007, 2009, 2011, 2012, 2013
29	Sand mine	Medium	51.1166	10.1677	1996, 2000, 2002, 2004, 2006, 2008, 2011, 2012, 2013
30	Natural cave	Medium	50.6852	8.2132	1995, 1997, 2005, 2007
31	Sand mine	Medium	51.2148	10.0778	2003, 2004, 2009, 2011, 2013
32	Mine shaft	Dry	50.1016	7.9153	2011
33	Mine shaft	Medium	50.3864	8.0706	2013
34	Mine shaft	Medium	50.5896	8.6391	2008
35	BB tunnel	Dry	50.3302	9.6025	2014
36	Mine shaft	Wet	50.8598	9.7555	2001, 2003, 2007, 2011
37	Sand mine	Medium	51.2150	10.0790	1994, 2003, 2004, 2009, 2011, 2013
38	Mine shaft	Moist	50.0609	7.7813	2002, 2005
39	Mine shaft	Wet	51.1151	9.0086	2001, 2004, 2005, 2008, 2013, 2014
40	Mine shaft	Moist	51.3177	9.3952	2003, 2006, 2012
41	Natural cave	Moist	51.2325	8.9012	2003, 2004, 2005, 2007, 2008, 2010, 2013, 2014
42	Mine shaft	Moist	50.3665	8.6317	2011
43	Mine shaft	Wet	50.5168	9.5344	2001, 2003, 2004, 2005, 2007, 2008, 2010, 2011, 2013, 2014
44	Mine shaft	Moist	50.9963	8.5831	2003, 2004, 2005, 2008, 2011, 2012, 2013, 2014
45	Mine shaft	Medium	50.1555	8.0817	2011
46	Sand mine	Dry	51.3843	8.9929	2001
47	Mine shaft	Moist	50.2259	8.2693	2005
48	BB tunnel	Dry	50.9569	9.8046	2000, 2002, 2005, 2008, 2009, 2011, 2012, 2013
49	Natural cave	Medium	50.5162	8.3752	2001, 2002, 2004
50	Mine shaft	Moist	49.6714	8.8525	2005
51	Mine shaft	Wet	50.0075	7.9601	2007
52	Natural cave	Moist	51.3209	9.8542	2007

**Table 2:** Primers and probes used (modified after Rudolf et al. 2013).

Name of primer	Sequence
PipF	5′-GCGGCCAAATATTGAGACTT-3’
PipR	5′-CGTCCTCAAACATCCAGACA-3’
TorrF	5′-GACACAGGACGACAGAAA-3’
TorrR	5′-GCCTACGCAACTACTAAA-3’

Name of Probe	Sequence
PipPipProbe	5′-GCTTCGGTGAAGGTTTGTGT-3’
PipMolProbe	5′-TGAACCCTCCAGTAAGGTATCAACTAC-3’
TorrProbe	5′-CGATGATGCCTGTGCTACCA-3’

### Effects of precipitation and temperature

In order to investigate effects of temperature and precipitation conditions on these hibernating populations we performed a generalized linear model (GLM). Since the exact numbers of observed individuals were counted only up to 20 mosquitos in the original data collection we considered the number of hibernating mosquitos as a categorical variable of three classes: f = few for counts between 1 and 10 individuals found within the subterranean shelter; s = several for counts between 11 and 20 individuals; and m = many for counts more than 20 individuals. For this analysis we only considered hibernating mosquitoes found in the winter months (December to February).

To refine data quality and to reduce spatial autocorrelation we removed repeated samples from the same or nearby underground sites (within a radius of 600 m). Among all data from sampling sites within a certain area and of the whole time period from 1991 to 2014 we chose only one sample at random but favoring a sampling date early in winter (i.e. December > January > February) in order to minimize the effect of potential die-off of the mosquitoes during winter. This procedure resulted in 390 samples when only taking one sample per cave into account and finally 271 samples when removing additional caves within a radius of 600 m.

As explaining variables, we considered temperature and precipitation during spring (i.e. March, April, May), Summer (i.e. June, July, August) and Fall (i.e. September, October, November). These variables were only little intercorrelated (see Table 1 in the supplementary). Additionally, we accounted for the sampling month during winter (coded as December = 1, January = 2 and February = 3) and altitude. The altitude was taken into account as we used the regional averages of temperature and precipitation recorded for the State of Hesse by the Deutscher Wetterdienst (DWD = German weather service) as explaining variables in the GLM. Thus altitude was included in our model to account for the effect of decreasing temperature in higher altitudes. We assume that weather could have a different effect on mosquito abundance in different elevation (e.g. an extremely warm summer may have a positive effect in higher altitudes, while the differing temperature in lower altitudes is detrimental to mosquitoes). The inclusion of the sampling month was carried out due to the assumption that the number of mosquitoes in the caves tends to decrease over the winter months. Whether the number of mosquitoes in the caves decreases over the winter months will be examined below. The analysis was performed in R^41^ with package VGLM^42,43^.

### Effects of surroundings

Since it is assumed that the number of mosquitos decreases over winter, the mosquito abundance was compared between the winter months as well. We tested for significant differences in the mosquito abundance frequencies between winter sampling month by means of a chi squared test.

Since we assume that a decrease in mosquito abundance may be influenced by cave parameters, we perform the test separately for source material and cave moisture. The main rock type of the shelter or of the walls and ceilings was recorded and shelters were divided into two categories, acidic or alkaline, according to the surrounding rock types and their influence on the pH of water. During site visits, the underground sites were also characterized by moisture level (very dry, dry, humid, wet, constant flow). Due to the paucity of extremes, the categories very dry/dry and constant flow/wet were combined. With a Chi square test, we tested for significance between the frequencies of recorded abundances of hibernating mosquitoes (few, several, many) and winter months (December to February, with GraphpadPrism 8.02^44^) in the respective parameters and presented them as stacked bar charts.

Additionally, we performed further analysis on the surroundings of sampling sites. Different cave zones were also recorded whereby each mosquito specimen, as well as the counting or frequency estimation was attributed to a zone. Caves with several zones inhabited by mosquitos were also included in the calculation in the corresponding categories.

The surrounding environment of the caves was characterized to reveal potential associations with the number of mosquitos (categories based on numbers of individuals found and their frequency in the data set). For each cave, the percentages of different land cover types (using the Corine Land Cover data^45^) were calculated (using ArcGIS Version 10.7^46^) within the surrounding 200 m, 400 m, 800 m and 1600 m. The Land Cover data was compiled into four groups (coniferous forest (CLC category 312), broadleaf forest (CLC 311), anthropogenic (CLC categories 111, 112, 121, 122, 142), agriculture (CLC categories 211, 222, 231, 242, 243). Data that could not be effectively assigned to any category (less than 50% cover of a category) was omitted. These datasets were also tested for significance with a Chi squared test (GraphpadPrism 8.02^44^) in the respective parameters and presented as stacked bar charts.

## Results

### Genetic species composition

The molecular species identification shows a sympatric occurrence of *Cx. p. pipiens* and *Cx. torrentium* in most of the cavernous habitats while there were no clear differences in species composition when comparing different sampling years (results not shown). Shown are the species proportions of the samples over the years (min years of sampling per cave = 1, max sampling per cave = 13) (Fig. 1). Numbers of *Cx. p. pipiens* and *Cx. torrentium* caught were of similar size and distribution while the subspecies *Cx. p. molestus* occurred very rarely in our sampling (Pools with: only *Cx. torrentium*: 183 (25%), only *Cx. p. pipiens*: 135 (19%), both: 397 (55%), both and *Cx. p. molestus*: 8 (1%)). Our records also confirmed other mosquito species in Hessian caves: *Aedes cinereus*/*geminus* (1 female), *Aedes rossicus* (67 female,1 male), *Anopheles maculipennis* s.l. (3 female), *Anopheles* *marteri* (5 female), *Culiseta annulata* (204 female, 2 male).

### Effects of precipitation and temperature

We first displayed temperature and precipitation conditions (yearly mean temperature and mean precipitation during the active phase from April through September) together with the observed mosquito abundances in the caves during winter months (Fig. 2). In comparatively hot and dry years such as 2003, a low percentage of caves with high abundances can be observed, whereas high abundances could be observed in comparatively cool, humid years such as 2010 or 2007. This is not the case in 2008, for example. Therefore, in the GLM we do not consider the temperature and precipitation ratios averaged over the whole activity phase but by quarters.

**Figure 3:**
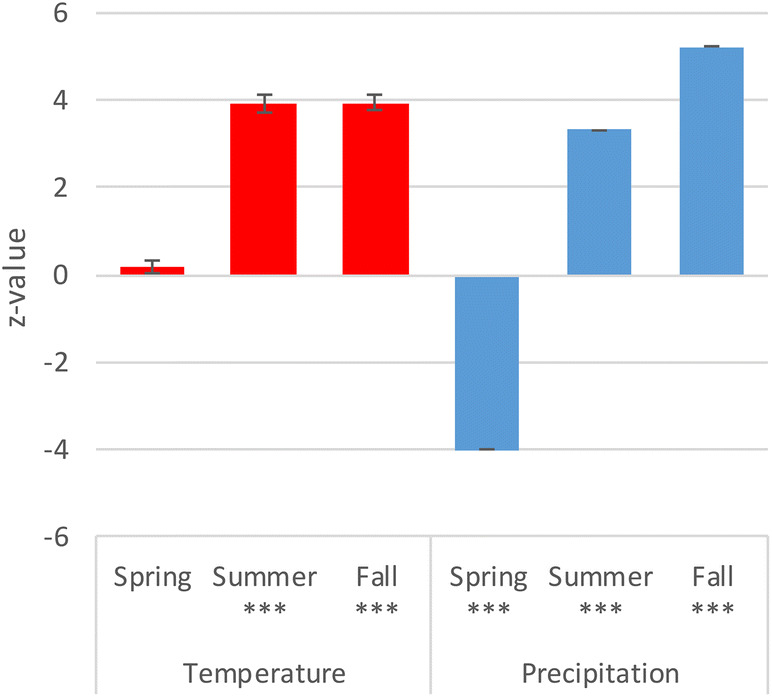
Effects of higher temperature and precipitation during activity and transition phases (March to November) on mosquito density in hibernacula during the hibernation phase (December through February). Depicted are the z-values of the GLM and their standard errors (p-values: *** < 0.001).

The GLM revealed that the abundance of hibernating mosquitoes is significantly affected by temperature in summer and fall as well as by precipitation of all three considered quarters (Supplementary). We illustrated the positive and negative effects of these variables in Fig. 3. Temperature in the spring months (March, April, May—T spring) had no significant effect while there was a significant increase during Summer (June, July, August) and Fall (September, October, November). Higher precipitation (P) had a significant negative effect during Spring and a significantly positive effect during Summer and Fall. A higher Altitude had a significant positive effect on the abundance as well (Supplementary model 1).

### Effects of surroundings

**Figure 4:**
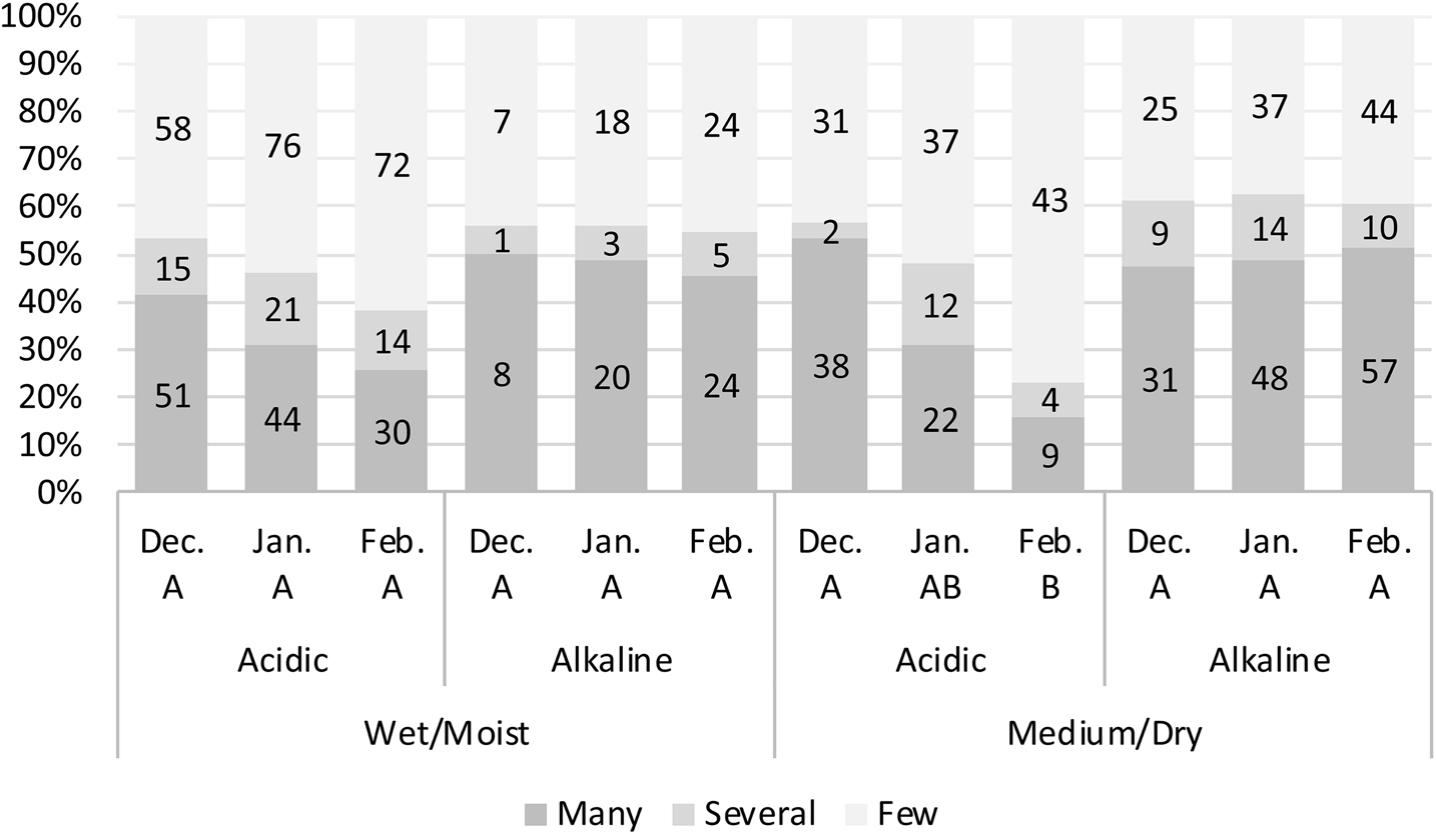
Comparison of mosquito abundance within hibernacles of alkaline and acidic surrounding rock combined with humidity levels. Statistical significance is symbolized with A, B and C, where non-matching letters are significantly different (Chi Square: 61.9, 22 df, p = 0.0002; corrected min./max. value A against B: p = 0.0004/0.0016). Y-Axis: composition of categories within the sampling. The numbers in the bar graphs show the absolute frequency of categories in the respective years. Categories: many: > 20 individuals, several: 10–20 individuals, few: 1–10 individuals found within the subterranean shelter.

There was a significant decrease in mosquito abundance during the winter months in subterranean environments surrounded by acidic rock composition in both, wet and dry underground shelters. On the other hand, the density of mosquitos within hibernacula classified as alkaline did not change during the winter months. The difference was strongest within the group of medium and dry underground shelters (Fig. 4).

A relationship between the abundance of mosquitos and the stated moisture levels of the underground habitats could not be established.

**Figure 5:**
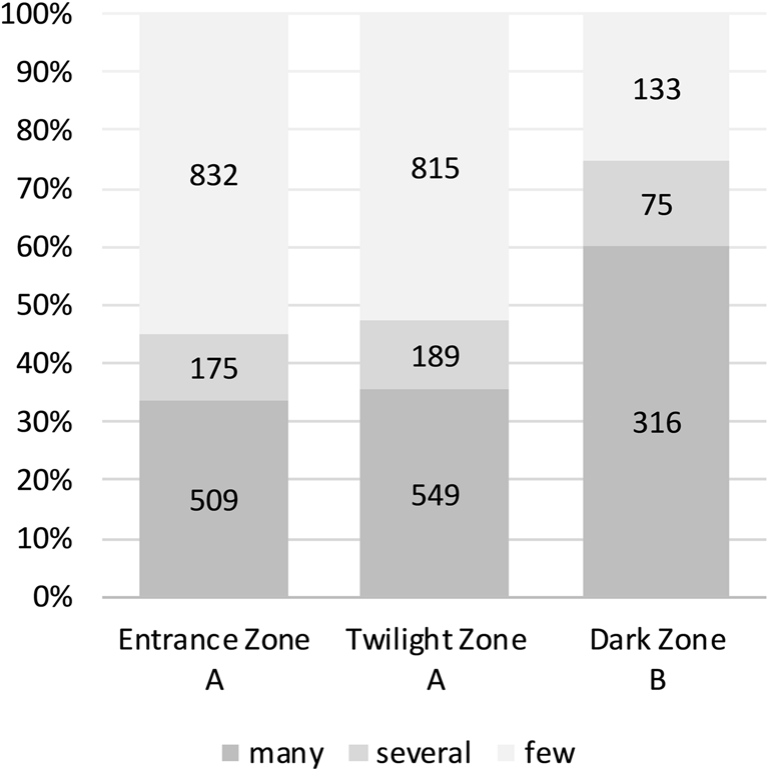
Comparison of mosquito abundance of the different depth zones within cavernous habitats during the hibernation period. Statistical significance is symbolized with A and B, where non-matching letters are significantly different (Chi Square: 154.3, 4 df, p < 0.0001; corrected value A against B: p < 0.0001). Y-Axis: composition of categories within the sampling. The numbers in the bar graphs show the absolute frequency of categories in the respective years. Categories: many: > 20 individuals, several: 10–20 individuals, few: 1–10 individuals found within the cave.

There is a significant difference between the number of mosquitos in the entrance and twilight zones compared to the dark zone (Fig. 5) in subterranean shelters. Dark zones seem to be favored be hibernating mosquitoes.

**Figure 6:**
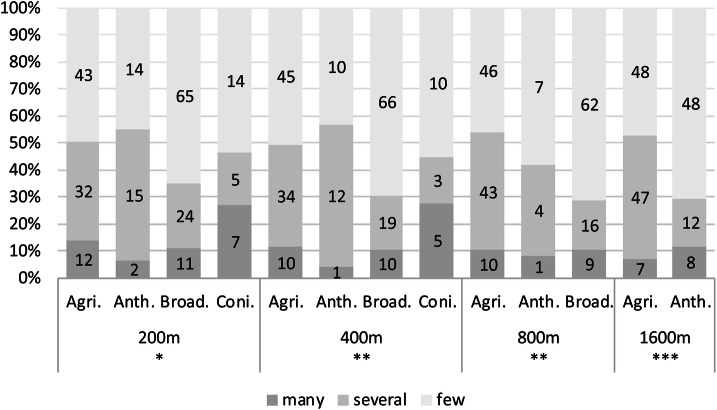
Comparison of mosquito abundance and different main land cover categories surrounding hibernacles in four different radiuses during the hibernation period. Abbreviations: Agri., agriculture and pastures; Anth., anthropogenic and urban; Broad., broadleaved forest; Coni., coniferous forest. p-values and Chi square statistics: 200 m: 0.02, 14.67, 6 df, Anth. against Coni.: 0.03, Anth. against Broad.: 0.03; 400 m: 0.004, 18.99, 6 df, Anth against Coni: 0.02, Anth. against Broad.: 0.007, Agri. against Broad.: 0.01; 800 m: 0.007, 14.8, 4 df, Agri. against Broad.: 0.007; 1600 m: 0.0007, 14.61, 2 df, Agri against Anth.: 0.0007. Omitted data from adjusted sample set of 263 sampling points: 200 m: 7%, 400 m: 14%, 800 m: 25%, 1600 m: 35%. Y-Axis: composition of categories within the sampling. The numbers in the bar graphs show the absolute frequency of categories in the respective years. Categories: many: > 20 individuals, several: 10–20 individuals, few: 1–10 individuals found within the subterranean shelter.

In addition, we considered the land cover characteristics of the surroundings of the underground sites. We found significant differences of the abundances of overwintering mosquitos between subterranean shelters surrounded by Broadleaf Forest and Conifer Forest and agricultural areas and Anthropogenic-purposed land (i.e. urbanization) (Fig. 6).

## Discussion

### Spatial patterns

No spatial pattern of species composition was detected in the subterranean shelters located in Hesse. However, the co-occurrence of the two species *Cx. p. pipiens* and *Cx. torrentium* was confirmed (Fig. 1). Similar results were shown by Rudolf et al.^40^ with a sample set of mosquitos collected in Germany above ground. For the State of Hesse, they found more *Cx. torrentium* than *Cx. p. pipiens* and no samples of *Cx. p. molestus*. Werblow et al.^47^ detected a general pattern of fewer *Cx. torrentium* than *Cx. pipiens*. According to Hesson et al.^14^, *Cx. torrentium* and *Cx. pipiens* occur sympatrically with more *Cx. torrentium* north of the Alps. In Central Europe and Austria, distributional patterns of both species are very similar^48–50^. Overall, it seems that *Cx. torrentium* and *Cx. p. pipiens* occur equally abundant in central Germany.

We could detect one hybridization event in our sampling. One pool of two collected specimens were molecularly flagged for all three subspecies. Although several authors already suggested hybridization between *Cx. p. pipiens* and *Cx. p. molestus*^51–53^, we were not able to determine which two out of the three sampled species hybridized. Since their genetic differences are overall very small^22,24^, it is questionable whether these two species should be distinguished as different subspecies. The authors suggested that the verification of gene flow between both forms allows for two different interpretations: They could be two genetically distinct forms, which converge and hybridize where their distribution areas are overlapping. Otherwise, *Cx. p. molestus* might just be a biotype that originated from *Cx. p. pipiens,* and the two of them are not yet reproductively isolated whereby similarities in behavior and physiology suggest hybridization. This suggests that *Cx. p. molestus* is a biotype of *Cx. pipiens* and, at least in Germany, relatively rare. Amara Korba et al.^23^ could prove the stenogamy and autogamy of *Cx. p. molestus* in 78.6% of their cases albeit this result does not account for all of the individuals. Another study revealed no prey choice preferences amongst birds and mammals regarding *Cx. p. pipiens* and *Cx. p. molestus*^16^. Overall, our findings corroborate those of other studies and point not to a picture of *Cx. p. molestus* as a subterranean subspecies that feeds on cave-dwelling mammals, but rather, to it being a biotype of *Cx. pipiens* that potentially has a shifted niche towards smaller enclosures like tree cavities or burrows of different animals that can also adapt to a surface habitat. If the two forms were only ecological varieties of one species, mixed forms should not be classified as hybrids.

The complete dataset contains 8750 *Culex* mosquitos, all collected within subterranean habitats. Of these samples, only 22 were male *Culex* mosquitos (collected in the months from May to December), of which 12 samples were classified as *Cx. torrentium* and 10 as *Cx. p. pipiens,* a finding which is supported by another study^54^. In light of the very low numbers of males and the lack of *Cx. p. molestus* or hybrid males, and since hibernating females of *Cx. p. molestus* collected in the subterranean shelters matched the percentage of those found by aboveground sampling, the surveyed Hessian *Cx. p. molestus* show no sign of homodynamy. Similar results confirm our assumption that *Cx. p. molestus* is not an underground biotype of *Cx. p*. *pipiens* or that subterranean objects are particularly suitable for hybridization^54^.

All other species, except two, were collected between April and September within the subterranean shelters, and therefore, show no evidence of hibernation within subterranean shelters. Only *Culiseta annulata* and one specimen of *Anopheles maculipennis* were collected during the winter months, possibly using caves for hibernation. Compared to the other rarer mosquitos, higher individual numbers of *Cu. annulata* underscore this consideration.

### Effects of precipitation and temperature

We provide evidence that weather conditions during the previous activity phase as well as the time of sampling influence the abundance of species within the hibernacles in the following winter. Temperature in the Spring (March, April and May) had no significant effect. This can be explained by a previous finding that higher temperatures in March probably cause the mosquitos to exit their wintering grounds prematurely^55^, resulting in a negative effect that is cancelled out by the following two months.

Climate effects on mosquitos were studied from early May to mid-September in a north-western province in Italy^56^. Although the climate of northern Italy is not completely comparable with that of Hesse, partially similar patterns were observed. These results are somewhat different from our calculations whereby a higher temperature in Summer (June, July and August) as well as in the Fall (September, October and November) correlated with a significant increase of mosquitos within the hibernacles. Lack of information concerning Italian populations after September precludes further comparison. Higher temperatures in September potentially affect the behavior of mosquitos, which generally start migrating into shelters for the winter from the beginning of October. Higher temperatures in October and November could enable more mosquitos to find suitable shelter for the winter, which results in a positive effect for our “Fall” category. Our results show a negative effect in Spring and a positive one in Summer and Fall, corroborating a preference for higher precipitation that was reported before^56,57^. Another study detected a direct correlation between higher temperature and the number of mosquitos two weeks later^58^. Our calculation revealed a significant effect of sampling months on the number of mosquitos found inside the underground structure, i.e. the later the sampling in the winter, the fewer mosquitos were found inside the cave, which is corroborated by the study of Zittra et al.^54^.

### Effects of surroundings

A decrease of mosquitos in January and February was detected. Smaller numbers are most prominent in underground habitats classified as dry/medium and acidic (Fig. 4). A similar, although not significant pattern is visible in wet/humid, acidic habitats. An attenuation of the impact on mosquito numbers with regard to increased humidity can be explained by the fact that contact more water weakens the pH-lowering effect of the surrounding material. More acidic environments may simply not be conducive for mosquito hibernation whereby the mechanisms involved could merit further investigation.

Distribution within caves and the higher number of mosquitos in the deeper parts of the underground habitats was unexpected. It was previously assumed that mosquitos would hibernate mainly in the entrance and twilight zones^36^. There might be a possible tradeoff reflected in the results in that overwintering in the deeper zones might guarantee better shelter against freezing outside temperatures and higher survival rates.

The effective flight distance for *Culex* mosquitos throughout their life span is between 600 to 2000 meters^59,60^. For our analysis, we therefore set four different radiuses of 200, 400, 800 and 1600 m around the hibernation site to analyze land cover more closely. Overall, anthropogenic and agricultural surroundings have a positive effect on mosquito density within the hibernacles when compared to forests. Although coniferous forests in Germany are often monocultures for timber production that provide a suitable habitat for only a limited number of vertebrate species^61–63^, we could not detect a significant difference between the two forest types. The increased proportion of the “many” and “several” categories in agricultural and anthropogenic areas might be explained by the fact that fields and pastures are frequently used by large grazing animals that serve as hosts for adult mosquitos. Water troughs as well as car tires, commonly used as weights on tarpaulins, are ideal breeding grounds for mosquito larvae. Most settlements within the flight radius of the surveyed hibernacles are small clusters of single-family houses with large gardens, which often also contain many small collections of water in rainwater barrels, plant pots or buckets. These breeding options as well as proximity to humans, pets and birds provide excellent living conditions for mosquitos. Our findings of greater abundancies of species in hibernacles surrounded by anthropogenic and agriculturally influenced terrains is a common pattern found in urban habitats^15,48,50,57^.

## Conclusion

Germany and the State of Hesse lie in the temperate climate zone, where caves and other underground shelters offer an advantage for *Cx. pipiens* and *Cx. torrentium*, if not a necessity. Our study complements existing knowledge about the ecological requirements of this species complex. By using information about climate conditions and mosquito densities within caves the following winters, it might be possible to estimate which years witness a large mosquito density and thus create temporal pattern forecasts. The sites, their characteristics and surroundings are important for the occurrence of the species and create spatial patterns. Spatial and temporal patterns are particularly important for vector species as they allow the necessary precautions to be assessed and applied more quickly.

In summary, based on results of previous studies, we expected to find significantly higher proportions of *Cx. p. molestus* inside the caves, but our results indicate a similar species composition of the *Culex pipiens* complex as that found outside the caves. We did not find any male specimens of *Cx. p. molestus*, a fact that suggests that at least in our study area, *Cx. p. molestus* lacks permanent underground populations and does not reproduce in subterranean environments. Our results also show that for Hesse, the previous theory that mosquitos hibernate primarily in the entrance zones of caves should be re-evaluated. We assume that the frequency of mosquitos within the caves is determined by the frequency of mosquitos on the surface. This assumption is supported by our results that the climate conditions during the activity phase have a significant effect on the frequency of hibernating mosquitos. Therefore, we argue that the number of hibernating mosquitos could be taken as a proxy for the overall density of the mosquito population but would require further investigation. The availability of suitable hibernation sites ensures the continuation of the species in the following year. Dependent on cave parameters, we could detect a decrease in the abundance of overwintering mosquitos during the winter months.

## Supplementary information

Supplementary file

## References

[1] Vinogradova EB (2000). Culex pipiens pipiens mosquitoes. Taxonomy, distribution, ecology, physiology, genetics, applied importance and control.

[2] Linnaeus C (1758). Systema naturae Vol. 1. No. part 1.

[3] Forskål, P. *Flora Ægyptiaco-Arabica sive descriptiones plantarum quas per ægytum inferiorem et arabiam felicem detexit, illustravit Petrus Forskål. Post mortem auctoris edidit Carsten Niebuhr* (1775).

[4] Martini R (1925). Zwei bemerkenswerte Culiciden von einem eigenartigen Biotop. Int Rev Hydrobiol.

[5] Say T (1823). Descriptions of dipterous insects of the United States. J. Acad. Nat. Sci. Philadelphia.

[6] Coquillett DW (1898). Report on a collection of Japanese Diptera, presented to the U.S. national museum by the Imperial University of Tokyo. Proc. US Natl. Museum.

[7] Meigen, J. W. & Wiedemann, C. R. W. Aussereuropäische Zweiflügelige Insekten / beschrieben von Christ. Rud. Wilh. Wiedemann ; als Fortsetzung des Meigenischen Werkes.; 10.5962/bhl.title.14603 (1828).

[8] Barr AR (1967). Occurrence and distribution of the *Culex pipiens* complex. Bull. World Health Organ..

[9] Hubálek Z, Halouzka J (1999). West Nile Fever–a reemerging mosquito-borne viral disease in Europe. Emerg. Infect. Dis..

[10] Lundström JO (1999). Mosquito-borne viruses in western Europe: a review. J. Vector Ecol..

[11] Hayes CG (2001). West Nile virus: Uganda, 1937, to New York City, 1999. Ann. N. Y. Acad. Sci..

[12] Hubálek Z (2008). Mosquito-borne viruses in Europe. Parasitol Res.

[13] Werblow A, Bolius S, Dorresteijn AWC, Melaun C, Klimpel S (2013). Diversity of Culex torrentium Martini, 1925—a potential vector of arboviruses and filaria in Europe. Parasitol Res.

[14] Hesson JC (2014). The arbovirus vector *Culex torrentium* is more prevalent than *Culex pipiens* in northern and central Europe. Med. Vet. Entomol..

[15] Becker N (2010). Mosquitoes and Their Control.

[16] Gomes B (2013). Feeding patterns of *molestus* and *pipiens* forms of Culex pipiens (Diptera: Culicidae) in a region of high hybridization. Parasites Vectors.

[17] Andreadis TG (2012). The contribution of *Culex pipiens* complex mosquitoes to transmission and persistence of West Nile virus in North America. J. Ame. Mosquito Control Assoc..

[18] Lõhmus M, Lindström A, Björklund M (2012). How often do they meet? Genetic similarity between European populations of a potential disease vector *Culex pipiens*. Infect. Ecol. Epidemiol..

[19] Harbach RE, Harrison BA, Gad AM (1984). *Culex (Culex) molestus* Forskal (Diptera: Culicidae): neotype designation, description, variation, and taxonomic status. Proc Entomol Soc Wash.

[20] Knight KL (1951). A Review of the *Culex pipiens* complex in the Mediterranean Subregion (Diptera, Culicidae). Trans. R. Entomol. Soc. Lond..

[21] Kamura T, Bekku H (1959). Studies on the *Culex pipiens* group of Japan. IV. Ecological studies on the Nagasaki molestus. Endemic Dis. Bull. Nagasaki Univ..

[22] Kent RJ, Harrington LC, Norris DE (2007). Genetic Differences Between *Culex pipiens* f. *molestus* and *Culex pipiens pipiens* (Diptera: Culicidae) in New York. J. Med. Entomol..

[23] Amara Korba R (2016). Ecological differentiation of members of the *Culex pipiens* complex, potential vectors of West Nile virus and Rift Valley fever virus in Algeria. Parasites Vectors.

[24] Vinogradova EB, Shaikevich EV (2007). Morphometric, physiological and molecular characteristics of underground populations of the urban mosquito *Culex pipiens* Linnaeus f. molestus Forskål (Diptera: Culicidae) from several areas of Russia. Eur Mosq Bull.

[25] Sulaiman S, Service MW (1983). Studies on hibernating populations of the mosquito *Culex pipiens* L. in southern and northern England. J. Nat. History.

[26] Huang S (2009). Genetic Variation Associated with Mammalian Feeding in *Culex pipiens* from a West Nile Virus Epidemic Region in Chicago Illinois. Vector-Borne Zoonotic Dis..

[27] Denlinger DL, Armbruster PA (2014). Mosquito diapause. Annu. Rev. Entomol..

[28] Onyeka JOA, Boreham PFL (1987). Population studies, physiological state and mortality factors of overwintering adult populations of females of *Culex pipiens* L. (Diptera: Culicidae). BER.

[29] Spielman A (1964). Studies on Autogeny in *Culex pipiens* Populations in Nature. I. Reproductive isolation between autogenous and anautogenous populations. Am. J. Hygiene.

[30] Harbach RE, Harrison BA, Gad AM (1984). Culex (Culex) molestus Forskal (Diptera: Culicidae): neotype designation, description, variation and taxonomic status. Proc Entomol.

[31] Harbach RE, Dahl C, White GB (1985). *Culex (Culex) pipiens* Linnaeus (Diptera: Culicidae): Concepts, type designations, and description. Proc. Entomol. Soc. Wash..

[32] Merdić E, Vujičić-Karlo S (2005). Two types of Hibernation of *Culex pipiens* complex (Diptera: Culicidae) in Croatia. Entomol. Croatia.

[33] Kjærandsen J (1993). Diptera in mines and other cave systems in southern Norway. Entomologica Fennica.

[34] Badino G (2004). Cave temperatures and global climatic change. IJS.

[35] Barr RA (1967). Ocurrence and distribution of the Culex pipiens Complex. Bull. World Health Organ..

[36] Gunn J (2004). Encyclopedia of caves and karst science.

[37] *Höhlen. Verborgene Welten.* 1st ed. (Primus-Verl., Darmstadt, 2008).

[38] Buffington JD (1972). Hibernaculum choice in *Culex Pipiens*. J. Med. Entomol..

[39] Thomson RCM (1938). The reactions of mosquitoes to temperature and humidity. Bull. Entomol. Res..

[40] Rudolf M (2013). First nationwide surveillance of *Culex pipiens* complex and *Culex torrentium* mosquitoes demonstrated the presence of *Culex pipiens* biotype *pipiens/molestus* hybrids in Germany. PLoS ONE.

[41] R Core Team (2013). R. A Language and Environment for Statistical Computing.

[42] Yee TW, Wild CJ (1996). Vector generalized additive models. J. R. Stat. Soc. Ser. B (Methodol.).

[43] Yee TW (2015). Vector Generalized Linear and Additive Models: with an Implementation in R.

[44] GraphPad Software. *GraphPad Prism* (La Jolla California USA).

[45] Copernicus Land Monitoring Service. *Corine Land Cover Data* (European Environment Agency (EEA), 2019).

[46] Systems E (2019). ArcGIS Desktop.

[47] Werblow A (2014). Population structure and distribution patterns of the sibling mosquito species *Culex pipiens* and *Culex torrentium* (Diptera: Culicidae) reveal different evolutionary paths. PLoS ONE.

[48] Weitzel T, Jawień P, Rydzanicz K, Lonc E, Becker N (2015). *Culex pipiens* s.l. and *Culex torrentium* (Culicidae) in Wrocław area (Poland): occurrence and breeding site preferences of mosquito vectors. Parasitol Res.

[49] Lühken R (2015). Physico-chemical characteristics of *Culex pipiens* sensu lato and *Culex torrentium* (Diptera: Culicidae) breeding sites in Germany. J. Med. Entomol..

[50] Zittra C (2016). Ecological characterization and molecular differentiation of *Culex pipiens* complex taxa and *Culex torrentium* in eastern Austria. Parasites Vectors.

[51] Fonseca DM (2004). Emerging vectors in the *Culex pipiens* complex. Science.

[52] Gomes B (2009). Asymmetric introgression between sympatric *molestus* and *pipiens* forms of *Culex pipiens* (Diptera: Culicidae) in the Comporta region, Portugal. BMC Evol. Biol..

[53] Andreadis TG, Huang S, Molaei G (2011). Reexamination of *Culex pipiens* hybridization zone in the Eastern United States by Ribosomal DNA-based single nucleotide polymorphism markers. Am. J. Trop. Med. Hyg..

[54] Zittra C, Moog O, Christian E, Fuehrer H-P (2019). DNA-aided identification of *Culex* mosquitoes (Diptera: Culicidae) reveals unexpected diversity in underground cavities in Austria. Parasitol. Res..

[55] Ciota AT, Matacchiero AC, Kilpatrick AM, Kramer LD (2014). The Effect of Temperature on Life History Traits of *Culex* Mosquitoes. J. Med. Entomol..

[56] Rosà R (2014). Early warning of West Nile virus mosquito vector: climate and land use models successfully explain phenology and abundance of *Culex pipiens* mosquitoes in north-western Italy. Parasites Vectors.

[57] Zittra C (2017). Landscape structure affects distribution of potential disease vectors (Diptera: Culicidae). Parasites Vectors.

[58] Paz S, Albersheim I (2008). Influence of warming tendency on Culex pipiens population abundance and on the probability of West Nile fever outbreaks (Israeli Case Study: 2001–2005). EcoHealth.

[59] Verdonschot PFM, Besse-Lototskaya AA (2014). Flight distance of mosquitoes (Culicidae): A metadata analysis to support the management of barrier zones around rewetted and newly constructed wetlands. Limnologica.

[60] Ciota AT (2012). Dispersal of Culex mosquitoes (Diptera: Culicidae) from a wastewater treatment facility. J. Med. Entomol..

[61] Otto H-J (1994). Waldökologie.

[62] Brockerhoff EG, Jactel H, Parrotta JA, Quine CP, Sayer J (2008). Plantation forests and biodiversity: oxymoron or opportunity?. Biodivers. Conserv..

[63] Ellenberg H, Leuschner C, Dierschke H (2010). Vegetation Mitteleuropas mit den Alpen in ökologischer, dynamischer und historischer Sicht.

