# Supplementary material for: An investigation of hibernating members from the Culex pipiens complex (Diptera, Culicidae) in subterranean habitats of central Germany
Source: Sci Rep. 2020 Jun 24;10:10276. doi: 10.1038/s41598-020-67422-7 (PMC7314823; doi:10.1038/s41598-020-67422-7)
Supplement: Supplementary file 1 — Supplementary file [file 41598_2020_67422_MOESM1_ESM.docx]

**An investigation of hibernating members from the *Culex pipiens* complex (Diptera, Culicidae) in subterranean habitats of central Germany**

Dorian D. Dörge^1*^, Sarah Cunze^1^, Henrik Schleifenbaum^1^, Stefan Zaenker^3^, Sven Klimpel^1,2^

1 Institute for Ecology, Evolution and Diversity, Goethe-University, Max-von-Laue-Str. 13, Frankfurt/Main D-60439, Germany

2 Senckenberg Biodiversity and Climate Research Centre, Senckenberg Gesellschaft für Naturforschung, Senckenberganlage 25, Frankfurt/Main D-60325, Germany

3 Hesse Federation for Cave and Karst Research, Königswarter Str. 2a, Fulda D-36039, Germany

*Corresponding author: Dorian D. Dörge ([Doerge@bio.uni-frankfurt.de](mailto:Doerge@bio.uni-frankfurt.de))

**Supplementary**

**GLM**

Call:

vglm(formula = kat_abundance ~ Sampling_month + ALT + T_spring + T_summer + T_autumn + P_spring + P_summer + P_autumn, family = propodds,data = data)

Pearson residuals:

Min 1Q Median 3Q Max

logitlink(P[Y>=2]) -2.1768 -0.7952 -0.3474 0.8649 3.513

logitlink(P[Y>=3]) -0.9634 -0.4797 -0.2127 -0.1481 9.068

Coefficients:

Estimate Std. Error z value Pr(>|z|)

(Intercept):1 -25.662914 5.300776 -4.841 1.29e-06 ***

(Intercept):2 -27.643408 5.338626 -5.178 2.24e-07 ***

Sampling_month -0.283285 0.166155 -1.705 0.088205 .

ALT 0.002348 0.001027 2.285 0.022287 *

T_spring 0.027347 0.140432 0.195 0.845598

T_summer 0.875952 0.222809 3.931 8.45e-05 ***

T_autumn 0.675482 0.171157 3.947 7.93e-05 ***

P_spring -0.019442 0.004848 -4.010 6.07e-05 ***

P_summer 0.012547 0.003785 3.315 0.000916 ***

P_autumn 0.020985 0.004006 5.238 1.62e-07 ***

---

Signif. codes: 0 ‘***’ 0.001 ‘**’ 0.01 ‘*’ 0.05 ‘.’ 0.1 ‘ ’ 1

Names of linear predictors: logitlink(P[Y>=2]), logitlink(P[Y>=3])

Residual deviance: 465.2279 on 532 degrees of freedom

Log-likelihood: -232.6139 on 532 degrees of freedom

Number of Fisher scoring iterations: 5

Warning: Hauck-Donner effect detected in the following estimate(s):

'(Intercept):1'

Exponentiated coefficients:

Sampling_month ALT T_spring T_summer T_autumn P_spring P_summer P_autumn

0.7533051 1.0023503 1.0277248 2.4011613 1.9649800 0.9807461 1.0126265 1.0212065

**Table 1 – Correlation between temperature and precipitation**

| Pearson correlation coefficient | T_spring | T_summer | T fall | P_spring | P_summer | P_ fall |
| --- | --- | --- | --- | --- | --- | --- |
| T_spring | 1 | -0,11903653 | 0,0249715 | -0,3872084 | 0,42348521 | 0,62853121 |
| T_summer | -0,11903653 | 1 | -0,01219597 | 0,28669576 | -0,36982509 | 0,41026577 |
| T_fall | 0,0249715 | -0,01219597 | 1 | 0,18596725 | 0,04577676 | 0,6161362 |
| P_spring | -0,3872084 | 0,28669576 | 0,18596725 | 1 | -0,36003086 | -0,00517906 |
| P_summer | 0,42348521 | -0,36982509 | 0,04577676 | -0,36003086 | 1 | 0,12850578 |
| P_fall | 0,62853121 | 0,41026577 | 0,6161362 | -0,00517906 | 0,12850578 | 1 |
